# Frequent Detection of Latent Tuberculosis Infection among Aged Underground Hard Coal Miners in the Absence of Recent Tuberculosis Exposure

**DOI:** 10.1371/journal.pone.0082005

**Published:** 2013-12-02

**Authors:** Felix C. Ringshausen, Albert Nienhaus, Anja Schablon, José Torres Costa, Heiko Knoop, Frank Hoffmeyer, Jürgen Bünger, Rolf Merget, Volker Harth, Gerhard Schultze-Werninghaus, Gernot Rohde

**Affiliations:** 1 Department of Respiratory Medicine, Hannover Medical School, Hannover, Germany; 2 Institute for Health Services Research in Dermatology and Nursing, University Medical Center Hamburg-Eppendorf, Hamburg, Germany; 3 Faculty of Medicine, University of Porto, Alameda Professor Hernâni Monteiro, Porto, Portugal; 4 Department of Pneumology, Allergology, and Sleep Medicine, University Hospital Bergmannsheil, Ruhr-University Bochum, Bochum, Germany; 5 Institute for Prevention and Occupational Medicine of the German Social Accident Insurance, Institute of the Ruhr-University Bochum (IPA), Bochum, Germany; 6 Institute for Occupational and Maritime Medicine (ZfAM), University Medical Center Hamburg-Eppendorf, Hamburg, Germany; 7 Department of Respiratory Medicine, Maastricht University Medical Center, Maastricht, The Netherlands; National Institute of Infectious Diseases, Japan

## Abstract

**Background:**

Miners are at particular risk for tuberculosis (TB) infection due to exposure to silica dust and silicosis. The objectives of the present observational cohort study were to determine the prevalence of latent TB infection (LTBI) among aged German underground hard coal miners with silicosis or chronic obstructive pulmonary disease (COPD) using two commercial interferon-gamma release assays (IGRAs) and to compare their performance with respect to predictors of test positivity.

**Methods:**

Between October 2008 and June 2010, miners were consecutively recruited when routinely attending pneumoconiosis clinics for an expert opinion. Both IGRAs, the QuantiFERON®-TB Gold In-Tube (QFT) and the T-SPOT®.TB (T-SPOT), were performed at baseline. A standardized clinical interview was conducted at baseline and at follow-up. The cohort was prospectively followed regarding the development of active TB for at least two years after inclusion of the last study subject. Independent predictors of IGRA positivity were calculated using logistic regression.

**Results:**

Among 118 subjects (mean age 75 years), none reported recent exposure to TB. Overall, the QFT and the T-SPOT yielded similarly high rates of positive results (QFT: 46.6%; 95% confidence interval 37.6–55.6%; T-SPOT: 61.0%; 95% confidence interval 52.2–69.8%). Positive results were independently predicted by age ≥80 years and foreign country of birth for both IGRAs. In addition, radiological evidence of prior healed TB increased the chance of a positive QFT result fivefold. While 28 subjects were lost to follow-up, no cases of active TB occurred among 90 subjects during an average follow-up of >2 years.

**Conclusions:**

Considering the high prevalence of LTBI, the absence of recent TB exposure, and the currently low TB incidence in Germany, our study provides evidence for the persistence of specific interferon-gamma responses even decades after putative exposure. However, the clinical value of current IGRAs among our study population, although probably limited, remains uncertain.

## Introduction

Exposure to silica dust and silicosis are well-established risk factors for the development of active tuberculosis (TB). Both silicosis and TB are prevalent and concomitant diseases in many parts of the world, particularly where mining is still a major industry [1-6]. Furthermore, it is increasingly recognized that COPD is not only a sequela of pulmonary TB, but may also contribute to its development [5,7]. 

In Germany, the overall number of insured subjects in the mining industry as well as the annual incidence of TB in the general population have been decreasing steadily over the last decade (Table 1) [8]. Nevertheless, due to latency and long exposure periods, new cases of silicosis and associated TB (silicotuberculosis) are still occurring (Table 1). Since silicosis continues to progress even after the cessation of exposure, silicotic subjects are often at an advanced age at the time of diagnosis. 

**Table 1:** Study background: numbers of insured subjects and recognized cases of the occupational diseases silicosis and silicotuberculosis, Germany, 2000–2009^*a*^.

	**Number of insured subjects in the mining industry**		**Number of recognized silicosis cases**		**Number of recognized silicotuberculosis cases**	
**Year**	**Mining total**	**Hard coal mining^*b*^**	**Mining total**	**Hard coal mining^*b*^**	**Mining total**	**Hard coal mining^*b*^**
2000	118,770	55,707	1,200	1,027	20	15
2001	107,779	47,800	1,158	1,022	31	23
2002	105,722	45,661	970	873	21	18
2003	99,502	42,173	854	771	20	15
2004	92,772	40,134	866	756	18	13
2005	87,759	35,592	721	636	10	7
2006	81,595	32,473	550	447	13	9
2007	78,224	27,950	395	335	13	9
2008	75,646	24,840	320	243	10	9
2009	71,707	22,117	1,035	890	13	9
Total	919,476	374,447	8,069	7,000	169	127

^*a*^Source: Dr. W. Hummitzsch, Institution for Statutory Accident Insurance and Prevention in Resources and Chemical Industry, Sector Mining, Bochum, Germany, personal communication.

^*b*^Note: The numbers for hard coal mining apply to both underground and surface mining. In general, underground hard coal miners are concerned. The increase of recognized silicosis cases in 2009 is due to a change in the guideline for the diagnosis and expert opinion of minimal pneumoconiosis [24]. Due to long exposure times and latency, the presented numbers provide only rough estimates of the related risk of disease development.

In many countries the targeted screening of silicotic subjects and the preventive treatment of latent TB infection (LTBI) are essential components of TB control strategies [4,9-11]. In Germany, underground hard coal miners with suspected or already recognized occupational diseases silicosis or COPD are referred to outpatient pneumoconiosis clinics for an expert opinion and regularly reassessed for recognition, deterioration, and appropriate compensation within a statutory system. Since usually no routine screening for LTBI is performed among miners with silicosis or COPD, the prevalence of LTBI and the related risk of subsequent progression to active TB are unknown. However, the incidence of active TB may roughly be estimated from the respective annual numbers of insured (exposed) subjects in the mining industry and recognized cases of silicotuberculosis (Table 1; cumulative incidences: 33.9 (95% confidence interval (CI) 28.4–40.2) cases of silicotuberculosis per 100,000 hard coal miners vs. 8.2 (95% CI 8.12–8.24) TB cases per 100,000 in the general German population, Germany, 2000–2009) [12]. 

The rationale for refraining from screening this population is related to the limited efficacy of preventive therapy in patients with long-standing LTBI and silicosis [13,14], the fact that the risk of drug-induced hepatitis may outweigh that of developing active TB [13], and the well-known limitations of the tuberculin skin test (TST), which has been the only tool available for the immunodiagnosis of LTBI for almost a century, in particular the TST’s reduced sensitivity among the elderly [9-11,15,16]. 

In this respect, the two commercially available interferon-(IFN)-γ release assays (IGRAs) QuantiFERON®-TB Gold In-Tube (QFT; Cellestis/QIAGEN, Hilden, Germany) and T-SPOT®*.TB* (T-SPOT; Oxford Immunotec, Abingdon, UK) possess distinct advantages. These one-visit, *ex vivo* blood tests have high specificity and avoid sensitization, boosting of immune response, and cross-reactivity following Bacillus Calmette-Guérin vaccination and exposure to most non-tuberculous mycobacteria [17,18]. Several recent studies have demonstrated their superiority over the TST in elderly populations and their improved ability to predict progression to active TB in populations at risk like recent TB contacts, HIV-infected individuals, and silicotic subjects [10,11,16,19-21]. Accordingly, the national guidelines on the screening of TB contacts recommend the primary use of an IGRA among adults in Germany [22].

The primary objectives of the present study were to determine the prevalence of LTBI among a cohort of aged underground hard coal miners using two commercial IGRAs, the QFT and the T-SPOT, and to compare their performance with respect to predictors of test positivity. In addition, the cohort was prospectively followed regarding the development of active TB. 

## Methods

### Ethics statement

The present study received ethical approval by the institutional review board of the Ruhr-University Bochum, Germany (registration no. 3214-08). All participants gave their written and informed consent.

### Study setting, design, and population

This observational cohort study was conducted at two independent academic institutions with long-standing expertise in occupational and respiratory diseases, the University Hospital Bergmannsheil and the Institute for Prevention and Occupational Medicine of the German Social Accident Insurance (IPA), both located in Bochum, Germany.

Between October 2008 and June 2010, four chest and three occupational physicians prospectively and consecutively recruited eligible underground hard coal miners when routinely attending pneumoconiosis clinics for an expert opinion regarding the two occupational diseases (1) silicosis (including coal worker’s pneumoconiosis) or (2) COPD at the respective institutions. As a rule, all subjects had respiratory symptoms and either (1) proven exposure to respirable crystalline silica and profusion of rounded opacities at International Labor Office (ILO) category ≥1/1 [23] or (2) proven cumulative exposure to respirable dust equivalent to at least 100 (mg/m^3^)×years. 

**Figure 1:**
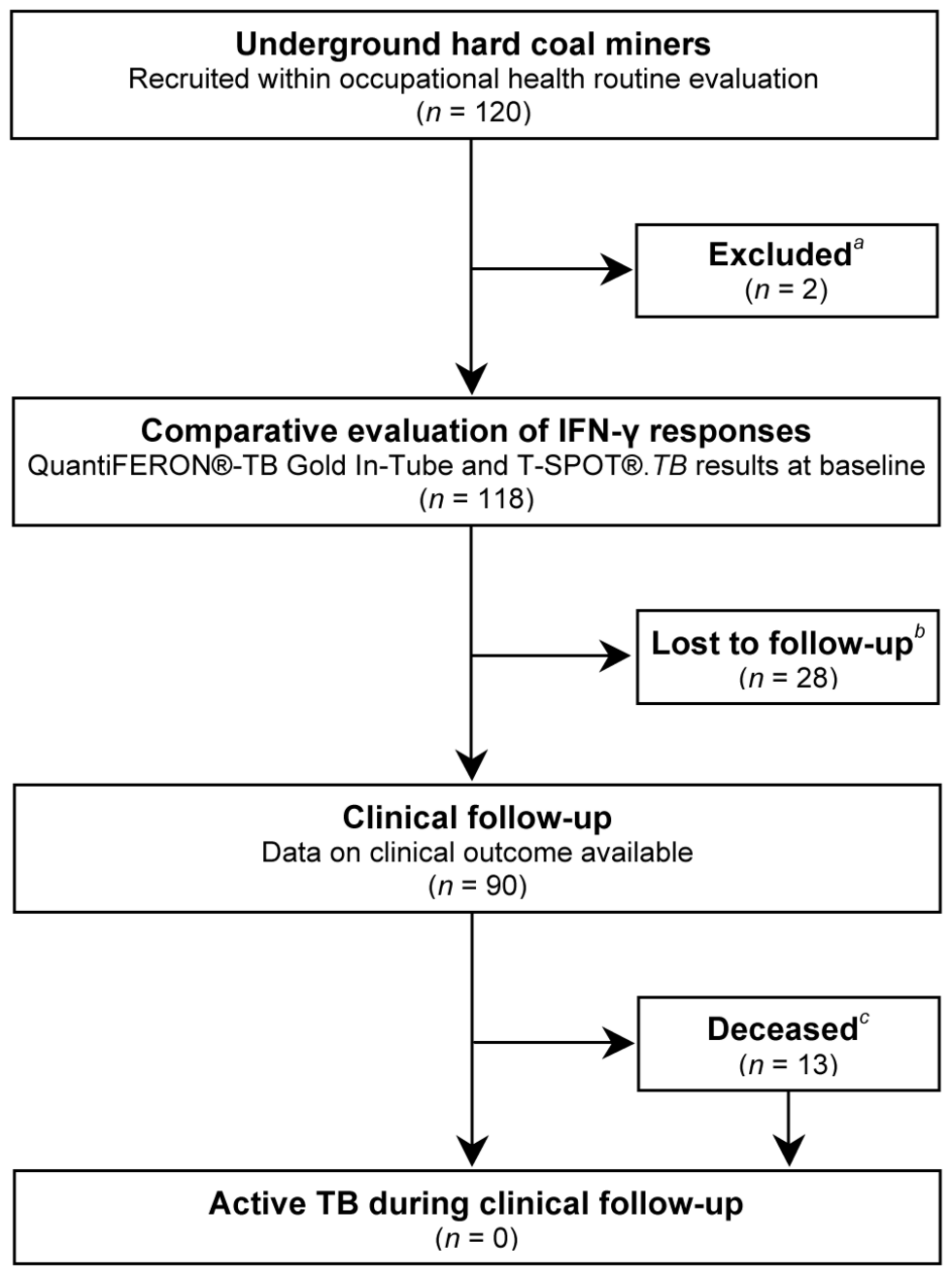
Study flow diagram. ^*a*^Reasons for study exclusion: no current chest imaging available (*n* = 2). ^*b*^Eight subjects had refused to participate in the follow-up interview. In 20 subjects the provided contact information had become invalid since study inclusion. ^*c*^Causes of death were malignancy (*n* = 5), cardiovascular diseases (*n* = 4), silicosis (*n* = 2), chronic obstructive pulmonary disease (*n* = 1), and pneumonia (*n* = 1).

Inclusion criteria were prior employment as an underground hard coal miner and appointment for an expert opinion regarding the occupational diseases silicosis or COPD at the instigation of the Institution for Statutory Accident Insurance and Prevention. Exclusion criteria were current treatment for LTBI or active TB. All subjects, or their closest relative, received a structured clinical interview at baseline and at follow-up. Both IGRAs were performed at baseline only (Figure 1). The study cohort was followed regarding the development of active TB for at least two years after the inclusion of the last study subject until June 2012, death, or the development of active TB, whichever occurred first. No preventive chemotherapy was offered to IGRA-positive individuals due to the fact that routine screening for LTBI was not performed among our study population, the undefined significance of a positive IGRA result among our study population, and the non-interventional nature of our study. However, all miners were subject to annual or biannual clinical and radiological routine reassessment according to German occupational safety and health (OSH) legislation. 

### Diagnostic methods

The structured interview and standardized questionnaire covered established occupational and non-occupational risk factors for recent TB infection, reactivation, and false-negative TB immune responses. Demographic and clinical data including prior and recent findings from chest radiographs, high-resolution computed tomography, and pulmonary function testing (PFT) were captured from occupational health records and from the current evaluation. Further routine work-up was conducted according to the national guideline on the diagnosis and expert opinion of the occupational disease silicosis [24]. Radiologic evidence of prior healed TB was assessed according to the American Thoracic Society [9] and the ILO [23]. If necessary, additional diagnostic procedures were performed at the discretion of the respective physician until a conclusive expert opinion could be established. The imaging studies were interpreted by experienced chest physicians or expert radiologists. 

The QFT measures IFN-γ (IU/ml) secreted by T-cells after stimulation with the *MTB*-specific antigens ESAT-6, CFP10, and TB7.7, whereas the T-SPOT measures IFN-γ-secreting spot-forming T-cells per well and 2.5×10^5^ peripheral blood mononuclear cells (SFC) using ESAT-6 and CFP10. Both IGRAs were manually performed in strict adherence to the manufacturers’ instructions and as previously described in detail [25]. IGRA results were considered positive if the QFT IFN-γ response of TB antigen minus nil control was ≥0.35 IU/ml (and ≥25% of the nil value) and, for the T-SPOT, if the number of SFCs was ≥6 SFCs for either ESAT-6 or CFP10 after subtracting the spot count of the nil control. 

### Definitions

Active TB: disease due to infection with MTB complex requiring a full course of chemotherapy and/or MTB detection by culture or by smear microscopy along with a confirmatory nucleic acid amplification test from the same specimen [8]. LTBI: persistent *Mycobacterium-tuberculosis*-(MTB)-specific T-cell responses in the absence of clinical evidence for TB disease, as assayed by the TST and/or current IGRAs [26]. Silicosis (occupational disease No. 4101, according to German ISH legislation): occupational history with proven exposure to respirable crystalline silica, profusion of rounded opacities at International Labor Office (ILO) category ≥1/1 [23], and compatible clinical signs and symptoms of disease. Silicotuberculosis (No. 4102): active pulmonary TB in a subject with recognized silicosis. COPD as an occupational disease (No. 4111): occupational history in underground had coal mining with proven cumulative exposure to respirable dust equivalent to at least 100 (mg/m^3^)×years, evidence of either airflow obstruction or hyperinflation on PFT, and compatible clinical signs and symptoms of chronic bronchitis and/or pulmonary emphysema [27]. Airflow obstruction: obstructive spirometry pattern with a forced expiratory volume in one second (FEV_1_)/forced vital capacity (FVC) ratio <0.7. Mild, moderate, severe, and very severe airflow limitation were defined according to the Global Initiative for Chronic Obstructive Lung Disease as a FEV_1_/FVC ratio <0.7 and a FEV_1_ ≥80% predicted, <80% and ≥50% predicted, <50% and ≥30% predicted, and <30% predicted, respectively [28]. Cumulative dust exposure was calculated as the airborne concentration of respirable dust at the workplace in mg/m^3^ multiplied with the number of years, in which the miner worked under usual labor conditions (assuming 220 eight-hour shifts per year).

### Statistical analysis

Pearson’s χ^2^ or Fisher’s exact tests were used for categorical data as appropriate. Normal distribution of continuous data was checked using the Kolmogorov-Smirnov test. Differences were determined with Student’s t-test, the Mann-Whitney U test, or the Wilcoxon test as appropriate. Cohen’s κ and Spearman’s ρ coefficients were calculated to assess agreement between dichotomous measures and correlation of continuous measures. Independent predictors of IGRA positivity were calculated using logistic regression. Variables were eligible for inclusion in logistic regression analysis if at least ten subjects with a positive IGRA result were within the respective category. All potential predictor or confounder variables of interest were entered simultaneously and model building was performed backward using the chance criteria for variable selection. Variables considered to be clinically significant were retained regardless of statistical significance [29]. There was no evidence of multicollinearity. Associations were described as adjusted odds ratio (OR) and 95% confidence interval (CI). P-values and 95% CI were calculated from Wald statistics and bootstrapping, respectively, with statistical significance set to p<0.05. Accordingly, differences were considered statistically significant if 95% CIs were not overlapping. 

The same technician performed repeat QFT enzyme-linked immunosorbent assays on the same set of aliquoted specimens for a random subset of 10 QFT assays on the same day as an additional quality control measure. QFT test-retest reproducibility was assessed using Cohen’s κ for qualitative QFT results and the intraclass correlation coefficient of continuous measures for quantitative QFT results. 

Due to the explanatory nature of our study and as the prevalence of LTBI among underground hard coal miners and among corresponding age groups of the general German population is unknown, no formal sample size calculation was done. Data analysis was performed using IBM SPSS Statistics, version 20 (IBM Corp., New York, NY). 

## Results

### Study population

All 120 consecutive miners who were invited to participate agreed to enroll. None of them was currently treated for LTBI or active TB. Two subjects were excluded from analysis because no chest imaging was available from the current evaluation (one experienced occupational physician considered repeat chest imaging unnecessary given an unchanged clinical and functional status and the availability of extensive prior chest imaging). A total of 118 males constituted the final study population (Figure 1). Table 2 summarizes the characteristics of the study population stratified by QFT and T-SPOT positivity. Mean age (± standard deviation [SD]) was 75 ± 6 (range, 55–89) years. The mean duration of working underground was 26 ± 9 years. The median start of working underground was the year 1951, while the median end was 1980 (interquartile range, 1949–1987). Age was significantly correlated with the year of starting employment in underground hard coal mining (ρ = -0.73; *P*<0.0001), but not with the overall duration of working underground (ρ = -0.073; *P* = 0.44). All subjects except one 56-year-old were retired, on average since the year 1987 (interquartile range, 1983–1991). Moderate airway obstruction was the predominant lung function pattern (mean FEV_1_/FVC ratio 62.7 ± 11.1, mean FEV_1_ 77.5 ± 22.8% predicted, mean FVC 89.2 ± 21.3% predicted). The mean cumulative dust exposure among subjects with COPD was 132 ± 31 100 (mg/m^3^)×years. Subjects with a history of prior TB were significantly more likely to have chest imaging compatible with prior healed TB (6/10; 60.0% vs. 20/108; 18.5%; *P* = 0.008). All study participants complained about at least one respiratory symptom. Ninety-two subjects (78%) complained about the combination of dyspnea on exertion (110/118; 93%), chronic cough (106/118; 90%), and chronic expectoration (96/118; 81%). None of the study participants reported known recent TB exposure or HIV infection. 

**Table 2:** Characteristics of the study population, stratified by QFT and T-SPOT positivity.

**Variables**		**Total**	**Positive** **QFT result**	**Positive** **T-SPOT result**
		***n* (%)**	***n* (% of total)**	***n* (% of total)**
Subjects, total		118 (100)	55 (46.6)	72 (61.0)
Age categorized				
	55–69 years	18 (15.2)	9 (50.0)	9 (50.0)
	70–79 years	73 (61.9)	27 (37.0)	40 (54.8)
	≥80 years	27 (22.9)	19 (70.4)	23 (85.2)
Reason for expert opinion				
	COPD	72 (61.0)	29 (40.3)	40 (55.6)
	Silicosis	46 (39.0)	26 (56.5)	32 (69.6)
Foreign country of birth^*a*^		18 (15.3)	13 (72.2)	14 (77.8)
Birth in high TB burden country^*b*^		3 (2.5)	3 (100)	3 (100)
Personal history of prior TB^*c*^		10 (8.5)	5 (50.0)	7 (70.0)
History of TB household exposure^*d*^		6 (5.1)	5 (83.3)	5 (83.3)
Comorbidities and risk factors*^e^*,*^f^*		65 (55.1)	28 (43.1)	37 (56.9)
	Diabetes mellitus	31 (26.3)	14 (45.2)	20 (64.5)
	Malignancy	17 (14.4)	6 (35.3)	9 (52.9)
	Chronic kidney disease	15 (12.7)	4 (26.7)	8 (53.3)
	Long-term steroid medication^*g*^	10 (8.5)	6 (60.0)	6 (60.0)
	Multiple	12 (10.2)	2 (16.7)	5 (41.7)
Inhaled corticosteroids		45 (38.1)	24 (53.3)	28 (62.2)
Smoking behavior				
	Current smoking^*h*^	16 (13.6)	11 (68.8)	12 (75.0)
	Ex-smoking^*h*^	80 (67.8)	11 (50.0)	11 (50.0)
	Never smoking	22 (18.6)	44 (45.8)	61 (63.5)
Pathological radiology finding(s)^*f*^		112 (94.9)	54 (48.2)	69 (61.6)
	Silicosis including PMF	63 (53.4)	29 (46.0)	39 (61.9)
	Emphysema	60 (50.8)	31 (51.7)	41 (68.3)
	Prior healed TB	26 (22.0)	18 (69.2)	20 (76.9)

^*a*^Turkey (*n* = 7), Poland (*n* = 6), Croatia (*n* = 1), and Hungary (*n* = 1); including countries with a high burden of TB.

^*b*^Annual TB incidence >50 per 100 000 population according to the World Health Organization [12]: Bosnia and Herzegovina, Latvia, and Russia (*n* = 1, each).

^*c*^Ten subjects had had prior TB between 1958 and 2008, on average 34 ± 17 years ago. Two subjects had had TB within the past decade and had completed treatment in 2001 and 2008 after 6 and 10 months of standard combination chemotherapy, respectively.

^*d*^Subjects with a history of household exposure to *Mycobacterium tuberculosis* had been exposed between 1940 and 1960, on average 57 ± 9 years ago.

^*e*^Conditions which account for an increased individual risk of recent TB infection, LTBI reactivation, and false-negative TB immune responses; in addition: underweight ≥10% (*n* = 3), status post gastrectomy (*n* = 3), chronic hepatitis B virus infection (*n* = 1), and sarcoidosis (*n* = 1).

^*f*^Multiple selections were possible.

^*g*^In the median, the oral steroid dose was equivalent to 5 mg prednisone (range 5–20 mg).

^*h*^On average, current smokers and ex-smokers had a smoking history of 27 ± 18 pack years.

Definition of abbreviations: COPD = chronic obstructive pulmonary disease; PMF = progressive massive fibrosis; QFT = QuantiFERON®-TB Gold In-Tube; TB = tuberculosis; T-SPOT = T-SPOT®.*TB*.

#### IGRA results and concordance between IGRAs

**Figure 2:**
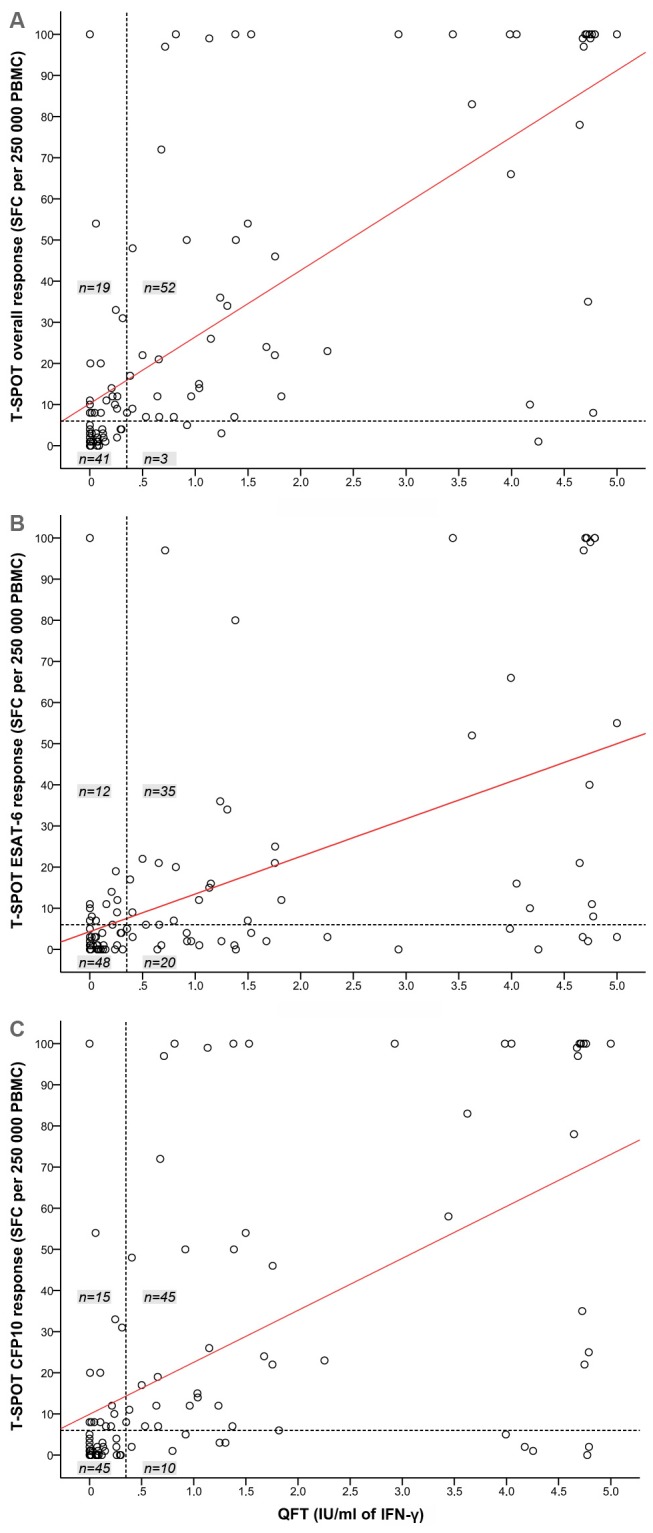
Distribution of quantitative T-SPOT and QFT responses. (A) T-SPOT overall, (B) T-SPOT ESAT-6, and (C) T-SPOT CFP10 responses plotted against the quantitative QFT IFN-γ response. T-SPOT responses >100 SFC are shown as 100 SFC. QFT IFN-γ responses >5.0 IU/ml are shown as 5.0 IU/ml. The dashed horizontal and vertical lines represent the diagnostic cut-offs of 0.35 IU/ml (QFT) and 6 SFC (T-SPOT). The red diagonal line represents the regression line. IFN-γ: interferon-γ; PBMC: peripheral blood mononuclear cells; QFT: QuantiFERON®-TB Gold In-Tube; SFC: spot forming cells; T-SPOT: T-SPOT®.TB.

Positive results were more frequently observed for the T-SPOT (61.0%; 95% CI 52.2–69.8%) than for the QFT (46.6%; 95% CI 37.6–55.6%; Table 2). However, due to overlapping 95% CI this difference was not statistically significant. Overall, indeterminate results were observed in three subjects (3/118 subjects, 2.5%; 2/118 QFT, 1.7%; 1/118 T-SPOT, 0.8%), who all had an active malignant disease. Table 3 shows the agreement between QFT and T-SPOT results. There was strong overall agreement between dichotomous IGRA results (raw agreement = 80.9%; κ = 0.62; *P*<0.0001; Table 3). In addition, there were good correlations between continuous QFT and T-SPOT overall, CFP10, and ESAT-6 responses (ρ = 0.74; 0.65; and 0.57; respectively; *P*<0.0001, each; Figure 2). The median T-SPOT CFP10 response was higher than the ESAT-6 response in the total study population (7 vs. 3 SFCs; *P* = 0.002). Discordant IGRA results occurred in 25 of 118 subjects (21.2%). Of those, the majority were QFT-negative/T-SPOT-positive (19/25; 76.0%). Five of 10 subjects with a personal history of prior TB had discordant IGRA results. Of those, two were QFT-negative/T-SPOT-positive, while one subject was QFT-positive/T-SPOT-negative, one was QFT-indeterminate/T-SPOT-positive, and one QFT-negative/T-SPOT-indeterminate. There were no significant correlations between the years elapsed since diagnosis and the quantitative IFN-γ responses among subjects with a history of prior TB (ρ = 0.13; -0.15; -0.21; and -0.19; for QFT, T-SPOT overall, CFP10, and ESAT-6 responses, respectively; *P*>0.50, each). 

**Table 3:** Agreement between QFT and T-SPOT assay results (n = 115)^*a*,*b*^.

		**T-SPOT**		
		**Positive**	**Negative**	**Total**
**QFT**		***n* (%)**	***n* (%)**	***n* (%)**
	**Positive**	52 (45.2)	3 (2.6)	55 (47.8)
	**Negative**	19 (16.5)	41 (35.7)	60 (52.2)
**Total**		71 (61.7)	44 (38.3)	115 (100)

^*a*^Three subjects with indeterminate test results were excluded from analysis.

^*b*^Raw agreement = 80.9%; κ = 0.62; *P*<0.0001.

On average, QFT- and T-SPOT-positive subjects were significantly older than those with negative IGRA results (QFT: 77 ± 6 vs. 74 ± 7 years; *P* = 0.041; T-SPOT: 77 ± 6 vs. 74 ± 6 years; *P* = 0.018). However, there were no significant differences between QFT- and T-SPOT-positives and those with negative results with respect to duration of underground work, lung function (FEV_1_/FVC, FEV_1_, FVC), and pack years smoked among current and former smokers (data not shown).

### Predictors of IGRA positivity

Multivariable logistic regression analysis demonstrated that age ≥80 years and birth in a foreign country were strong predictors of positive results for both IGRAs (Table 4). In addition, radiological evidence of prior healed TB raised the likelihood of a positive QFT result fivefold. However, silicosis and current smoking did not predict IGRA positivity (Table 4).

**Table 4:** Predictors of QFT and T-SPOT positivity (n = 115)^*a*^.

**Variables**		**Unadjusted OR (95% CI)**	***P* value**	**Adjusted OR (95% CI)**	***P* value**
**Positive QFT result**					
Age ≥80 years^*b*^					
	No	1	-	1	-
	Yes	4.0 (1.52–10.48)	0.005	5.8 (2.04–16.26)	<0.001
Reason for expert opinion					
	COPD	1	-	1	-
	Silicosis	2.1 (0.97–4.50)	0.059	1.8 (0.75–4.28)	0.19
Foreign country of birth^*b*^					
	No	1	-	1	-
	Yes	4.3 (1.32–14.24)	0.016	6.8 (1.91–24.0)	0.003
Current smoking					
	No	1	-	1	-
	Yes	3.5 (1.04–11.74)	0.043	3.5 (0.92–13.60)	0.066
Radiological evidence of prior healed TB^*b*^					
	No	1	-	1	-
	Yes	4.4 (1.59–12.07)	0.004	5.0 (1.69–14.98)	0.004
**Positive T-SPOT result**					
Age ≥80 years^*b*^					
	No	1	-	1	-
	Yes	6.4 (1.83–23.40)	0.004	8.1 (2.22–29.21)	0.001
Reason for expert opinion					
	COPD	1	-	1	
	Silicosis	1.85 (0.83–4.11)	0.13	1.5 (0.63–3.55)	0.36
Foreign country of birth^*b*^					
	No	1	-	1	-
	Yes	3.4 (0.91–12.44)	0.070	4.8 (1.26–18.07)	0.022
Current smoking					
	No	1	-	1	
	Yes	2.8 (0.74–10.47)	0.13	2.6 (0.65–10.65)	0.18
Radiological evidence of prior healed TB					
	No	1	-	1	-
	Yes	2.9 (0.98–8.30)	0.055	3.0 (0.96–9.15)	0.059

^*a*^Three subjects with indeterminate test results were excluded from logistic regression analysis.

^*b*^Variable included in final logistic regression model building.

### Follow-up and clinical outcome

Follow-up data were available for 90 of 118 (76.3%) study participants. Of those, 13 subjects (14.4%) deceased during the follow-up period (Figure 1). Among those deceased, IGRA results were comparable to the total study population with follow-up data available: 42 (46.7%), 56 (62.2%), and 19 (21.1%) of the total study population, and five (38.5%), seven (53.8%), and three (23.1%) of the deceased subjects had positive QFT, positive T-SPOT, and discordant results, respectively. Current expert opinion resulted in the subsequent diagnosis of lung cancer in two subjects. None of the participants was diagnosed with active TB at baseline or during the follow-up period of more than two years (mean 889 ± 297 days).

### Quality assessment

All assays met the manufacturers’ quality standards. One hundred and fifteen of 118 (97.5%) and 117 of 118 (99.2%) mitogen (= positive) controls showed strong responses >1.0 IU/ml and >20 SFCs for the QFT and the T-SPOT assay, respectively. In addition, QFT test-retest reproducibility was assessed on the same set of aliquoted specimens for a random subset of 10 QFT assays and demonstrated that results were reproducible and highly reliable for dichotomous (raw agreement 100%, κ = 1.0, *P*<0.0001) and continuous measures (intraclass correlation coefficient 0.980, 95% CI 0.923–0.995).

## Discussion

Both IGRAs yielded generally high rates of positivity, with the highest rates among the most advanced age group. However, no cases of active TB were observed during follow-up. In the absence of recent TB exposure, these findings indicate that T-cell mediated adaptive immune responses to MTB may persist even decades after initial infection. Thus, the added clinical benefit of IGRAs, though probably limited, remains uncertain among subjects with a high pretest probability of remote LTBI in a TB low-incidence country.

Data on the performance of IGRAs among subjects with silicosis are exceedingly limited [10,11]. A recent study from Hong Kong was the first to assess the performance of an IGRA, the T-SPOT, in the targeted screening for LTBI among aged silicotic subjects [10]. In this study, the overall rate of positive T-SPOT results of 64% was similar to our rate of 61%. In a follow-up study, Leung and colleagues demonstrated that a positive T-SPOT, but not a positive TST result predicted the subsequent development of active TB among 17 of 308 (5.5%) silicotic subjects after an average follow-up of 458 days [11]. In contrast, we detected no cases of active TB after an average follow-up of 889 days. In our study, advanced age and foreign origin increased the chance for a positive result for both IGRAs about five- to eightfold, while in the study by Leung et al. a positive T-SPOT result was only predicted by (current or former) smoking, but not by age [11]. In this respect, our findings are in accordance with previous studies on the prevalence of LTBI among German health care workers, which found age and foreign origin to be strong predictors of QFT positivity with rates well above 50% among subjects older than 60 years [30,31]. The finding that the radiographic evidence of prior healed TB predicted a positive QFT result is in line with a recent study from Korea, which found the QFT to be associated with radiographic lesions suggesting prior healed TB [16].

The present study has strengths and some inherent limitations. It evaluated two commercial IGRAs in a population at risk for LTBI as well as active TB in a head-to-head comparison and included a follow-up of more than two years. However, an inevitable limitation is the absence of a gold standard for the diagnosis of LTBI. In addition, the background prevalence of LTBI in the general German population is unknown and we did not include age-matched silica dust unexposed controls. Moreover, the number of included subjects was limited, our study was not powered to formally calculate the positive predictive value of a positive IGRA result, and a considerable proportion of subjects were lost during follow-up (n = 28; 23.7%). Another noteworthy limitation is the fact that both IGRAs are unable to distinguish between sensitization due to either MTB or non-tuberculous mycobacteria like *M. kansasii* or *M. marinum*, which share the same genes for ESAT-6 and CFP10 and have been reported to cause pulmonary infections among miners and silicotic subjects [1,11,32]. In addition, we were unable to account for a healthy worker “survivor” effect through which miners with an increased susceptibility for active TB and those who were unable to maintain LTBI due to insufficient immune responses may have been selected out of the study population over time. Furthermore, we are unable to fully exclude potential recall bias, although the information, which was provided by the study participants, was subject to extensive crosschecking with occupational health records. Lastly, it is important to note that our results may not apply to other populations and other epidemiological settings.

Our data argue against the waning of IFN-γ responses with time after initial infection with MTB as recently supposed by Mori and colleagues [33]. We recently demonstrated that the chance of a persistently positive MTB-specific IFN-γ response among health care workers increases with age [34]. It has been supposed that LTBI represents a variety of different immunological phenotypes, which the currently available IGRAs are unable to define with a single result [35,36]. Given the absence of recent TB exposure among our study population along with the steadily declining and currently low incidence of TB in Germany, positive IGRA results among our cohort are likely to represent long-standing LTBI. Our finding that, despite the high prevalence of positive IGRA results in up to 70–85% among those within the very advanced age group, no cases of active TB were detected during follow-up, supports the hypothesis that individuals who have been persistently IGRA-positive for a long time may have a better prognosis than those with a recent conversion [35,36]. 

Compared to the Hong Kong silicotic cohort with a similarly high but not age-related rate of LTBI [11], our results highlight the importance of the present TB background prevalence with respect to the performance of current IGRAs at given sensitivity and specificity values and the prediction of subsequent TB disease [37]. Hence, the high prevalence of LTBI in our study may rather reflect a birth cohort effect with remote exposure at times of high TB prevalence than ongoing TB transmission [38]. Although LTBI detection rates were not significantly different between both IGRAs, the higher rate of positive T-SPOT results and, on the other hand, the fact that radiological evidence of prior TB was a predictor of a positive QFT result may point toward the differential characteristics of current IGRAs, with the T-SPOT possessing superior sensitivity and the QFT possessing superior specificity [17,18].

The present study has important implications. Firstly, despite the high prevalence of LTBI no cases of active TB were detected during follow-up. Apparently, current IGRAs may possess a comparatively limited benefit to clinical care in our study setting. However, given the limitations of our study, the exact value for the prediction of subsequent TB disease aged underground hard coal miners in a country with a low incidence of TB remains uncertain. Secondly, our results support the age dependence of LTBI prevalence in Germany, which may be due to the evolution from a high to a low TB burden country in the past century [38]. In many other countries with a currently low incidence of TB, the growing proportion of older adults represent a vulnerable population at risk for active TB, with lower treatment success due to increased TB case fatality and significant comorbidity [39,40]. Remarkably, in 2011 the overall age-specific incidence of TB in the general German population was highest among males aged 70 years and above (11 per 100,000 population) [8]. Because the majority of TB disease in older adults may arise from reactivation of LTBI [41], enhanced identification and preventive treatment of those who are most likely to progress to active TB are urgently required in order to decrease the incidence of TB and to prevent secondary spread to the community [40]. Promising strategies to improve the immunodiagnosis of LTBI with regard to differences in biological behavior and prognosis include the exploration of IFN-γ responses to specific MTB latency antigens [42] and differential cytokine profiles of MTB-specific T-cells by flow cytometry [43,44]. Finally, it should be mentioned that individual and epidemiological factors need to be considered when choosing the appropriate diagnostic tool for TB screening according to the specific setting, as the impact on successive results and clinical outcomes may be considerable. 

## Conclusions

The QFT and the T-SPOT assay yielded similarly high rates of positive results, which were further increasing with advancing age to up to 70–85% among subjects aged ≥80 years. However, as no subsequent cases of active TB were detected during the follow-up of more than two years, the clinical importance of these findings is currently unknown. Considering the high prevalence of LTBI, the absence of recent TB exposure, and the currently low incidence of TB in Germany, our study provides evidence for the longevity of MTB-specific IFN-γ responses. Improved diagnostic tests that are able to delineate recent from remote TB infection and more accurately define the risk of progression from remote infection to TB disease are needed before the general screening of aged populations with a high likelihood of LTBI and preventive treatment based on positive screening results can be recommended. 
